# Impact of the 2022 ambulatory blood pressure monitoring guidelines on blood pressure phenotypes in pediatric kidney transplant recipients

**DOI:** 10.1007/s00467-026-07241-6

**Published:** 2026-03-10

**Authors:** Shanthi Sree Balani, Ruchi Gupta Mahajan, Yashwanth Gollapudi, Anjalin Sebastian, Michael Evans, Sarah Javed Kizilbash

**Affiliations:** 1 Division of Pediatric Nephrology, University of Minnesota, 2450 Riverside Ave, Minneapolis, MN 55454 USA; 2 Levine Children’s Hospital Pediatric Nephrology & Hypertension, Wake Forest University School of Medicine, Charlotte, NC USA; 3 University of Virginia, Charlottesville, VA USA; 4 Clinical and Translational Science Institute, University of Minnesota, Minneapolis, MN USA

**Keywords:** Pediatric kidney transplantation, Ambulatory blood pressure monitoring, Hypertension, Pediatric hypertension, AAP hypertension guidelines

## Abstract

**Background:**

The American Academy of Pediatrics Clinical Practice Guidelines (AAPCPG) recommend 24-h ambulatory blood pressure monitoring (ABPM) for diagnosing hypertension (HTN) in pediatric kidney transplant recipients. The ABPM guidelines were updated in 2022 to incorporate the 2017 AAPCPG thresholds for those ≥ 13 years and exclude blood pressure loads. The effect of the 2022 update on ABPM phenotype and its association with end-organ damage in this population remains unexplored.

**Method:**

We retrospectively evaluated pediatric kidney transplant recipients (age < 22 years) who underwent 24-h ABPM for HTN surveillance between 1/2021 and 9/2024. We grouped ABPM phenotypes into two categories: HTN (masked, ambulatory, and 2014-specific severe ambulatory) and no HTN (normal, white-coat, and 2014-specific uncategorized). We assessed systematic differences between the two guidelines using McNemar’s test and agreement using Cohen’s Kappa coefficient.

**Results:**

Our cohort included 105 recipients. Compared to 2014, the 2022 guidelines identified more recipients with HTN (49.5% vs. 36.2%; McNemar’s p < 0.001); however, the overall agreement remained substantial (kappa: 0.73, 95% CI: 0.61, 0.86). Among the 10 recipients with elevated ambulatory arterial stiffness index (AASI), the 2022 guidelines identified 80.0% as abnormal compared to only 30.0% under the 2014 guidelines (McNemar’s p = 0.025), reflecting no significant agreement (Kappa: 0.19; 95% CI: -0.1, 0.49).

**Conclusion:**

Compared to the 2014 criteria, the 2022 guidelines identified a higher proportion of pediatric kidney transplant recipients with HTN and more effectively identified abnormalities in recipients with elevated AASI. Our findings indicate that 2022 guidelines are better aligned with markers of arterial stiffness and cardiovascular risk.

**Graphical Abstract:**

A higher resolution version of the Graphical abstract is available as Supplementary information

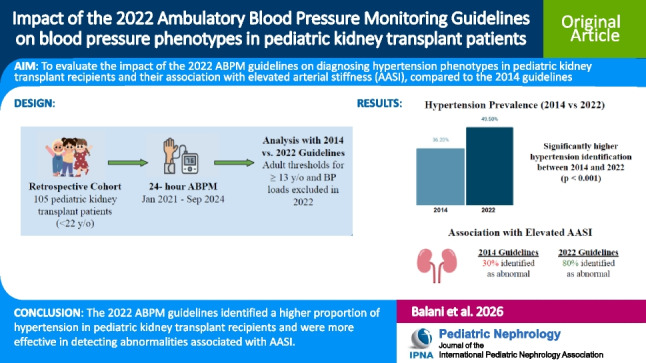

**Supplementary Information:**

The online version contains supplementary material available at 10.1007/s00467-026-07241-6.

## Introduction

Kidney transplantation is the treatment of choice for children with kidney failure. However, despite improvements in patient and graft survival, transplantation is associated with significant morbidity and mortality. Hypertension is a prevalent comorbidity among pediatric kidney transplant recipients, with an estimated prevalence of 58–89% [1]. It is associated with increased mortality, likely mediated by early-onset cardiovascular and cerebrovascular disease [1], and is an independent predictor of kidney graft failure across all age groups [1–3]. A timely and accurate diagnosis with effective management is necessary to optimize the long-term outcomes of kidney transplant recipients.

The American Academy of Pediatrics Clinical Practice Guidelines (AAP CPG) for Screening and Managing Hypertension in Children and Adolescents recommend 24-h ambulatory blood pressure monitoring (ABPM) to confirm hypertension in children with elevated clinic blood pressures [4, 5]. This recommendation is based on several proven benefits of ABPM compared to clinic measurements. ABPM provides more precise therapeutic targets for delaying the progression of chronic kidney disease (CKD) in children [6]. Furthermore, the ABPM-identified BP abnormalities in children are associated with end-organ damage, including alterations in carotid intima-media thickness (cIMT) and pulse wave velocity, left ventricular hypertrophy (LVH), and changes in neurocognition [7]. Studies in pediatric kidney transplantation also indicate that ABPM (2014 guidelines) outperforms clinic blood pressure measurements in predicting end-organ damage [8]. The AAP CPG recommends using ABPM in all kidney transplant recipients, irrespective of clinic blood pressure measurements, due to its ability to identify nocturnal and masked hypertension (MH) [4].

Until recently, ABPM was interpreted according to the 2014 ABPM guidelines, which recommend diagnosing hypertension based on mean ambulatory blood pressure (ABP) and ambulatory BP load (proportion of ABP readings > 95th percentile) [5]. However, the 2014 guidelines had limitations, frequently resulting in an 'unclassified' category for patients. Furthermore, the 2014 guidelines preceded the 2017 AAP CPG, which introduced static thresholds for hypertension for children ≥ 13 years (blood pressure < 130/80 mmHg), adapted from the 2017 American Heart Association (AHA) guidelines for adults. Therefore, the ABPM guidelines were revised in 2022 to incorporate the 2017 AAP CPG thresholds for hypertension for children 13 years and older. Additionally, based on the recent Chronic Kidney Disease in Children (CKiD) and the SHIP AHOY studies, which showed no association between BP loads and end-organ damage [2, 9, 10], BP loads were excluded from ABPM interpretation [11]. The ABP phenotypes of pediatric kidney transplant recipients under the 2022 ABPM guidelines have not been explored. Additionally, the association between the 2022 ABP phenotype and end-organ damage in pediatric kidney transplant recipients is unknown.

This study aimed to evaluate the ABP phenotype of pediatric kidney transplant recipients according to the revised 2022 ABPM guidelines and compare it to the phenotype based on the 2014 guidelines. The study also examined the association between ABP phenotype and end-organ damage. Given the static threshold for children aged 13 years and older, we hypothesized a higher prevalence of ambulatory hypertension under the 2022 guidelines compared to the 2014 guidelines, as the static thresholds are generally lower than the previous 95th percentile values. To our knowledge, this is the first pediatric study to characterize ABP in kidney transplant recipients using the new guidelines.

## Methods

The Institutional Review Board (IRB) at the University of Minnesota approved this study (STUDY00017370).

### Study population

We retrospectively identified all pediatric kidney transplant recipients (age < 22 years) who successfully completed 24-h ABPM for hypertension surveillance per the institutional protocol at the University of Minnesota between 1/2021 and 9/2024.

The ABPM protocol was introduced at our center in January 2020. It required all pediatric kidney transplant recipients with heights greater than 120 cm to undergo annual ABPM studies for the surveillance of hypertension starting 12 months post-transplant, regardless of their hypertension history.

### Study variables

We used data from a prospectively maintained, IRB-approved, solid organ transplant registry at the University of Minnesota. The transplant database contains post-transplant, peri-transplant, and minimal pre-transplant data on recipients and donors. Information on demographics, pre-transplant comorbidities, transplant surgery variables, post-transplant complications, and outcomes is captured via manual chart abstraction and electronic data transfer from the electronic medical record. The variables not available in the registry were abstracted directly from electronic medical records.

We examined the following variables: demographic characteristics, pre-transplant dialysis status (yes/no), donor source (living or deceased), body mass index (BMI) (calculated as weight in kilograms divided by height in meters squared), underlying cause of kidney failure, estimated glomerular filtration rate (eGFR) at the time of ABPM (calculated using the CKiD U25 (modified Schwartz) equation) [12] and ABP phenotype.

Echocardiograms were ordered per the physicians’ discretion, and those completed within 6 months of the ABPM study were included. Elevated left ventricular mass index (LVMI) was defined as LVMI > 95th percentile for age and sex [13].

Ambulatory arterial stiffness index (AASI) calculated by Spacelabs sentinel report was used and considered elevated if greater than 0.5 [14].

### ABP phenotype

The 2014 and the 2022 AHA pediatric ABPM guidelines are presented in Table 1 [5, 15]. Normotension was defined as normal clinic blood pressure and normal ABPM results. Ambulatory hypertension was defined as elevated clinic blood pressure and abnormal ABPM. White coat hypertension was defined as elevated clinic blood pressure but normal ABPM results. Masked hypertension was indicated by normal clinic blood pressure but abnormal ABPM results. We defined isolated nocturnal hypertension (INH) as normal daytime blood pressure but elevated nocturnal blood pressure, and isolated diastolic hypertension as elevated diastolic blood pressure but normal systolic blood pressure.

**Table 1:** Classification of hypertension based on 2022 updated criteria versus 2014 criteria

2022 ABPM guidelines					2014 ABPM guidelines		
Category	Clinic systolic or diastolic blood pressure*		Mean ambulatory SBP or DBP		Office BP	Mean ambulatory SBP or DBP	SBP or DBP Load %
	< 13 y of age	≥ 13 y of age	< 13 y of age	≥ 13 y of age			
Normal blood pressure	< 95th percentile	< 130/80	< 95th percentile OR adolescent cut points*	< 125/75 mmHg 24-h AND < 130/80 mmHg wake AND < 110/65 mmHg sleep	< 90%th percentile	< 95%th percentile	< 25
White coat hypertension	≥ 95th percentile	≥ 130/80			≥ 95th percentile	< 95%th percentile	< 25
Masked hypertension	< 95th percentile	< 130/80	≥ 95th percentile OR adolescent cut points*	≥ 125/75 mmHg 24-h OR ≥ 130/80 mmHg wake OR ≥ 110/65 mmHg sleep	< 95th percentile	> 95%th percentile	≥ 25
Ambulatory hypertension	≥ 95th percentile	≥ 130/80			> 95th percentile	> 95%th percentile	25–50
Prehypertension					> 95th percentile	< 95%th percentile	≥ 25
Severe ambulatory hypertension					> 95th percentile	> 95%th percentile	> 50

Under normal circumstances, BP drops by 10 to 20% during sleep, a phenomenon referred to as dipping. We defined ‘non-dipping’ or ‘blunted dipping’ as a < 10% decrease in systolic BP during sleep and defined ‘reversed-dipping’ as a higher mean systolic sleep BP compared to the mean wake BP [5].

We defined pre-existing hypertension as the use of antihypertensive agents for blood pressure regulation at the time of ABPM.

### Statistical analysis

Data are summarized as medians with interquartile ranges (IQRs) and proportions. We compared continuous and categorical variables between patients with and without BP abnormalities using the Wilcoxon rank sum and Fisher’s exact tests. We used logistic regression to examine the association between eGFR and hypertension. To compare the 2014 and 2022 guidelines, ABPM phenotypes were grouped into two categories: hypertension (masked, ambulatory, and 2014-specific severe ambulatory) and no hypertension (normal, white-coat, and 2014-specific uncategorized). Systematic differences in classification were assessed using McNemar’s test, and concordance was quantified using Cohen’s Kappa coefficient. These analyses were conducted for the total cohort and for a subgroup of patients with an elevated AASI, defined as > 0.5. Statistical analyses were performed using SAS software, version 9.4 (SAS Institute Inc., Cary, NC). A two‐sided P < 0.05 was considered statistically significant.

## Results

Our cohort included 105 recipients with a median duration since transplant at the time of ABPM of 5.26 years (IQR: 2.12, 8.81). At the time of the study, 76 recipients were on antihypertensive medications for existing hypertension, while 29 had no history of hypertension.

According to the 2022 guidelines, 52 recipients (49.5%) were identified as having blood pressure abnormalities (AH and MH); this included 12 recipients with new-onset hypertension and 40 with poorly controlled, pre-existing hypertension. When combined with the 36 recipients whose hypertension was well-controlled on medication, the overall prevalence of hypertension in our cohort was 83.8% (n = 88).

There was no significant association between eGFR and overall hypertension status (existing and new-onset) (OR: 0.99, 95%CI: 0.97, 1.03; p = 0.89), and hypertension was prevalent across all stages of CKD. The distribution of CKD stages and corresponding hypertension prevalence were as follows: Stage I (n = 6 total; 100% hypertensive), Stage II (n = 50; 80.0% hypertensive), Stage III (n = 43; 83.7% hypertensive), Stage IV (n = 4; 100% hypertensive), and Stage V (n = 2; 100% hypertensive). The majority of hypertensive recipients were in Stages II and III (45.4% and 40.9% of the hypertensive cohort, respectively).

### Baseline cohort characteristics

Table 2 presents the demographic and baseline clinical characteristics of pediatric kidney transplant recipients with and without blood pressure abnormalities on ABPM, according to the 2022 guidelines. Abnormal ABPM (AH, MH) was significantly more common in recipients older at transplant (median: 11.8 vs. 6.1; p = 0.002). Abnormal ABPM was also more prevalent in the group maintained on tacrolimus and mycophenolate compared to those on other maintenance immunosuppression combinations, however, the difference did not reach statistical significance (p = 0.06). We observed no differences between recipients with abnormal ABPM (n = 52) and those with normal ABPM (n = 53) regarding sex (p = 0.71), racial distribution (p = 0.48), donor source (p = 0.91), and the cause of kidney failure (p = 0.42). Similarly, there was no difference in CKD stage distribution between recipients with abnormal ABPM and those with normal ABPM (p = 0.56).

**Table 2:** Demographic and baseline characteristics by ambulatory hypertension per the 2022 guidelines

Variables	Ambulatory HTN on ABPM* N = 52	No ambulatory HTN on ABPM* N = 53	P value
Age at transplant, Median (IQR)	11.8 (6.5, 15.8)	6.1 (2.6, 12.5)	0.002
Age at ABPM, Median (IQR)	16.4 (14.0, 19.4)	14.5 (10.7, 16.4)	0.002
Sex: Male, n (%)	38 (73.1)	37 (69.8)	0.71
Race, n (%)			0.48
White Black Asian Native American	37 (71.1) 4 (7.7) 5 (9.6) 6 (11.5)	38 (71.7) 8 (15.1) 4 (7.5) 3 (5.7)	
Deceased donor transplant, n (%)	23 (44.2)	24 (45.3)	0.91
BMI percentile, Median (IQR)	80.8 (60.6, 94.7)	72.3 (46.4, 94.3)	0.61
Primary disease, n (%)			0.42
CAKUT Non-CAKUT	25 (51.0) 24 (49.0)	29 (59.2) 20 (40.8)	
CSA/MMF CSA/MMF/steroids CSA/Aza CSA/Aza/steroids other Tac/MMF Tac/MMF/steroids Tac/Aza Tac/Aza/steroids	2 (3.8) 3 (5.8) 2 (3.8) 1 (1.9) 2 (3.8) 23 (44.2) 9 (17.3) 4 (7.7) 6 (11.5)	0 (0.0) 1 (1.9) 1 (1.9) 5 (9.4) 10 (18.9) 16 (30.2) 6 (11.3) 4 (7.5) 10 (18.9)	0.06
CKD stage			0.56
CKD stage 1	3 (5.8)	3 (5.7)	
CKD stage 2	23 (44.2)	27 (50.9)	
CKD stage 3	23 (44.2)	20 (57.7)	
CKD stage 4	1 (1.9)	3 (5.7)	
CKD stage 5	2 (3.9)	0	

### ABP phenotype (2022 guidelines)

#### Entire cohort

Table 3 presents the ABP phenotype of the study cohort according to the 2022 guidelines. AH and MH were seen in 21% and 29.5% of the cohort, respectively. White coat hypertension, or white coat effect (in those with pre-existing hypertension), was observed in 7.6% of recipients, while isolated nocturnal hypertension and isolated diastolic hypertension were observed in 39.1% and 13.3%, respectively. Furthermore, 53.3% of the recipients displayed blunted dipping, and another 8.6% showed reverse dipping.

**Table 3:** Prevalence and characteristics of post-transplant ambulatory hypertension based on 2022 ABPM guidelines

Prevalence	All N = 105	On antihypertensive medication(s) at the time of ABPM* N = 76	No prior diagnosis of hypertension** N = 29
Ambulatory hypertension (abnormal ABPM and abnormal clinic BP)	21 (20.0%)	16 (21.1%)	5 (17.2%)
Masked hypertension (abnormal ABPM but normal clinic BP)	31 (29.5%)	24 (31.6%)	7 (24.1%)
Normal	45 (42.8)	31 (40.8)	14 (48.3)
White coat hypertension or white coat effect (normal ABPM but abnormal clinic BP)	8 (7.6%)	5 (6.6%)	3 (10.3%)
Isolated nocturnal hypertension	41 (39.1%)	29 (38.2%)	12 (41.4%)
Isolated diastolic hypertension	14 (13.3%)	10 (13.2%)	4 (13.8%)
Isolated systolic hypertension	11 (10.5%)	8 (10.5%)	3 (10.3%)
Blunted nocturnal dipping	56 (53.3%)	42 (55.3%)	14 (48.3%)
Reverse dipping	9 (8.6%)	6 (7.9%)	3 (10.3)

^*^Indicates hypertension control among those with a known history of hypertension

#### Recipients with pre-existing hypertension

AH was observed in 21.1%, and MH was seen in 31.6% of the recipients already on medications for hypertension, indicating poor control. Additionally, the white-coat effect was observed in 6.6% of recipients with pre-existing hypertension.

ABP phenotype per the 2014 guidelines is shown in Table 4. Nearly 6% were categorized as severe AH, and 15% remained unclassified, with only elevated blood pressure loads.

**Table 4:** Prevalence and characteristics of post-transplant ambulatory hypertension based on 2014 ABPM guidelines

Prevalence	All N = 105	On antihypertensive medication(s) at the time of ABPM* N = 76	No prior diagnosis of hypertension** N = 29
Ambulatory hypertension (abnormal ABPM and abnormal clinic BP)	9 (8.6%)	8 (10.5%)	1 (3.5%)
Masked hypertension (abnormal ABPM but normal clinic BP)	23 (21.9%)	18 (23.7%)	5 (17.2%)
Normal	44 (41.9%)	30 (39.5%)	14 (48.3%)
White coat hypertension or white coat effect (normal ABPM but abnormal clinic BP)	7 (6.7%)	4 (5.3%)	3 (10.3%)
Severe ambulatory	6 (5.7%)	4 (5.3%)	2 (6.9%)
Uncategorized – elevated loads	16 (15.2%)	12 (15.8%)	4 (13.8%)
Isolated nocturnal hypertension	32 (30.5%)	25 (32.9%)	7 (24.1%)
Isolated diastolic hypertension	13 (12.4%)	11 (14.5%)	2 (6.9%)
Isolated systolic hypertension	1 (0.95%)	1 (1.3%)	0 (0.0%)
Blunted nocturnal dipping	56 (53.3%)	42 (55.3%)	14 (48.3%)
Reverse dipping	9 (8.6%)	6 (7.9%)	3 (10.3)

^*^Indicates hypertension control among those with a known history of hypertension

### 2022 versus 2014 guidelines

Table 5 compares ABP phenotypes according to the 2022 vs. the 2014 guidelines.

**Table 5:** The 2022 versus the 2014 ambulatory blood pressure phenotypes

ABP phenotype according to the 2014 guidelines	ABP phenotype according to the 2022 guidelines				
	Ambulatory HTN (n)	Masked HTN (n)	Normal (n)	White coat HTN (n)	Total (n, %)
Ambulatory HTN (n)	9	0	0	0	9 (8.6%)
Masked HTN (n)	0	23	0	0	23 (21.9%)
Normal (n)	3	3	38	0	44 (41.9%)
Severe ambulatory HTN (n)	6	0	0	0	6 (5.7%)
Uncategorized—elevated loads (n)	3	5	7	1	16 (15.2%)
White coat HTN (n)	0	0	0	7	7 (6.7%)
Total (n, %)	21 (20.0%)	31 (29.5%)	45 (42.9%)	8 (7.6%)	105 (100.0%)

Compared to the 2014 guidelines, the 2022 guidelines identified a higher number of pediatric recipients with AH (20.0% (n = 21) vs. 14.3% (n = 15)) and MH (29.5% (n = 31) vs. 21.9% (n = 23)). Overall, the prevalence of hypertension was significantly higher using the 2022 criteria compared to the 2014 criteria (49.5% vs. 36.2%; McNemar’s p < 0.001). Despite the increased sensitivity of the newer guidelines, the overall agreement between the two standards remained substantial (kappa statistic: 0.73, 95% CI: 0.61, 0.86).

Of the 44 recipients with **normal** ABPM according to the 2014 guidelines, 6 recipients had abnormal ABPM (AH = 3, MH = 3) according to the 2022 guidelines. Additionally, of the 16 recipients who were uncategorized under the 2014 guidelines (elevated loads only), 3 were classified as AH and 5 as MH under the 2022 guidelines.

#### 2022 versus 2014 ABP phenotypes by age

Among recipients aged ≥ 13 years, the 2022 guidelines identified a higher proportion of AH (23.0% (n = 17) vs. 14.9% (n = 11)) and MH (33.8% (n = 25) vs. 23.0% (n = 17)) compared to the 2014 guidelines. This difference was primarily driven by recipients labeled as ‘uncategorized’ under the 2014 guidelines; of the 16 uncategorized recipients in the entire cohort, 13 (81.2%) were in the ≥ 13 age group. Under the 2022 guidelines, 8 (61.5%) of these 13 recipients were reclassified as AH (n = 3) or MH (n = 5). Overall, the prevalence of hypertension among recipients aged ≥ 13 years was significantly higher using the 2022 criteria compared to the 2014 criteria (56.76% vs. 37.84%; McNemar’s p < 0.001). Despite these differences, the overall agreement between the two guidelines in identifying hypertension for the recipients aged ≥ 13 years was substantial (kappa: 0.63, 95% CI: 0.47, 0.79).

In contrast, there was almost perfect agreement between the 2022 and 2014 guidelines for recipients aged < 13 years (kappa: 1.0, 95% CI: 1.0, 1.0). In this younger group, both guidelines identified 4 recipients with AH (3 with ambulatory and 1 with severe ambulatory under 2014) and 6 with MH. Furthermore, the three younger recipients who were uncategorized per the 2014 guidelines were all diagnosed as normal per the 2022 guidelines.

### Ambulatory hypertension and target organ injury

Of the 47 recipients who underwent echocardiogram, elevated LVMI was found in 17 (36.2%). The association between hypertension (existing and new-onset) according to the 2022 guidelines and elevated LVMI approached statistical significance (hypertension vs. no hypertension: 41.5% vs. 0.0%; p = 0.07). The association between hypertension (existing and new-onset) according to the 2014 guidelines and elevated LVMI was statistically significant (hypertension vs. no hypertension: 43.6% vs. 0.0%; p = 0.04).

Ten recipients had an elevated AASI. Among these, the 2022 guidelines identified 8 (80%) with abnormal ABPM phenotypes compared to only 3 (30%) under the 2014 guidelines (McNemar’s p = 0.025). Overall, the agreement between the 2014 and 2022 guidelines in identifying blood pressure abnormalities in recipients with elevated AASI was poor and statistically non-significant (Kappa: 0.19; 95% CI: −0.1, 0.49).

## Discussion

This is the first study to examine the prevalence of hypertension in pediatric kidney transplant recipients using the revised 2022 AHA guidelines and to compare the 2022 ABP phenotype with the 2014 ABP phenotype. The overall prevalence of hypertension using the 2022 guidelines was 84%, which is consistent with the previously reported prevalence in pediatric kidney recipients, ranging from 58 to 89% [1]. Using the 2022 criteria, we identified a significantly higher prevalence of AH and MH compared to the 2014 guidelines, particularly among recipients aged ≥ 13 years. We observed significant rates of MH (30%) and isolated nocturnal hypertension (35%), diagnoses that cannot be ascertained using clinic blood pressure measurements. Furthermore, the 2022 guidelines demonstrated greater sensitivity in identifying recipients with elevated arterial stiffness (AASI), underscoring the clinical utility of the updated criteria. Our study findings highlight the burden of hypertension in pediatric kidney transplant recipients and indicate the necessity of routine surveillance using 24-h ABPM.

Although there was a substantial agreement between the 2022 and 2014 guidelines in identifying hypertension (kappa: 0.73), the 2022 guidelines classified a higher proportion of recipients as hypertensive (p < 0.001). The 2022 guidelines captured every recipient identified as abnormal by the 2014 criteria, while also diagnosing an additional 11.4% with AH and 7.6% with MH. Notably, this included six recipients who had previously been classified as normal under the 2014 thresholds. These findings are similar to the 11% increase in AH reported in a Canadian study comparing the 2022 guidelines to the 2014 AHA and 2016 ESH guidelines in the general pediatric population [16]. The present study is the first study to evaluate this diagnostic shift in pediatric kidney transplant recipients specifically.

One of the significant updates in the revised 2022 ABPM guidelines was the recommendation to use static, adult-aligned blood pressure thresholds for adolescents aged 13 and older, consistent with the 2017 AAP CPG. Unsurprisingly, the shift in diagnostic criteria primarily affected the older age group; we observed a significantly higher proportion of AH and MH diagnoses in recipients ≥ 13 years using the 2022 guidelines. However, we observed no difference in AH or MH when we applied the two guidelines to the younger age group. These findings align with previous reports from the SHIP-AHOY and CKiD cohorts, which demonstrated significantly increased AH prevalence when using adult rather than pediatric cutoffs for older children (26.9% vs. 16.9% and 44% vs. 27%, respectively) [2, 17].

We observed a high prevalence of MH in our cohort, which increased from 21.9% under the 2014 guidelines to 29.5% using the 2022 guidelines. MH was also present in 23.6% of recipients already receiving antihypertensive therapy, indicating undertreated hypertension. Our results align with a study by Hamdani et al. [18], which identified MH in 38% of pediatric kidney transplant recipients using 2014 criteria. These findings are also consistent with a systematic review and meta-analysis of pediatric and young adult populations (ages 4–25 years), which found that while the prevalence of MH in the general population is 10.4%, it is significantly higher in children with CKD (RR: 2.44) and solid organ transplant recipients (RR: 2.34) [19]. The clinical significance of these findings is underscored by the association between MH and adverse outcomes, including lower estimated glomerular filtration rates [18], increased LVH, and elevated pulse wave velocity [19]. Since MH remains undetectable via clinic measurements and occurs at a high rate even in patients on antihypertensive medications, it is important for pediatric kidney transplant recipients to undergo routine ABPM testing.

The prevalence of isolated nocturnal hypertension in our cohort (35.2% overall and 36.8% for those on therapy) aligns with reported estimates of 30% to 40% in pediatric kidney transplant recipients [20]. Similar to MH, isolated nocturnal hypertension is clinically significant due to its association with cardiovascular abnormalities in patients with CKD, including LVH and increased cIMT [21]. Furthermore, the observed rates of blunted nocturnal dipping (61.9%) and reverse dipping (8.6%) are consistent with previous reports documenting abnormal dipping in 30–72% of transplanted children [1].

Isolated diastolic hypertension was seen in 12% of our cohort, with a large majority (84.6%; n = 11/13) of them representing poorly controlled hypertension. However, it may be an underestimation of the true prevalence as ABPM devices performed less reliably for diastolic compared to systolic measurements in validation studies [22]. The lack of variability in diastolic measurements in the ABPM normative data also suggests limited performance of ABPM for diastolic measurements [23].

We observed white coat hypertension in 7.6% of the entire cohort and white coat effect in 6.5% of those on antihypertensive therapy. The relatively lower prevalence of white coat hypertension in pediatric kidney recipients compared to the general pediatric population (30–40%) [24] is likely related to the overall high incidence of hypertension in transplant recipients. However, the white coat effect in 6.5% of patients with existing hypertension indicates the role of ABPM in preventing unnecessary escalation of antihypertensive therapy, which could result in clinically significant hypotension and potential graft hypoperfusion.

In our cohort, more than a third of recipients who underwent echocardiography demonstrated elevated LVMI, underscoring the substantial burden of cardiovascular end-organ damage in this population. The high prevalence of elevated LVMI in the hypertensive (pre-existing and new-onset) versus normotensive recipients suggests a clinically relevant relationship. Additionally, the majority of recipients with elevated AASI were classified as abnormal ABP under the revised 2022 ABPM guidelines compared with the 2014 guidelines, highlighting the updated recommendations' increased sensitivity in detecting vascular stiffness, which is closely linked to adverse cardiovascular remodeling. Our findings are consistent with those of a pediatric cross-sectional study conducted at a tertiary care outpatient hypertension clinic, which found that only the 2022 guideline significantly predicted elevated AASI (odds ratio: 2.4; p = 0.02) [16].

Our study had several limitations. Our sample size was small, limiting our ability to examine the association between ABP phenotype and LVH. Since the prevalence of hypertension varies with the length of posttransplant follow-up, our results may be an underestimation of the prevalence of abnormal blood pressure patterns within the first year posttransplant and an overestimation for patients who are several years posttransplant. Despite its limitations, this study is important as it describes the ABP phenotype using the newer guidelines and underscores the burden of hypertension in this population, emphasizing the need for routine ABPM to diagnose masked and isolated nocturnal hypertension.

To conclude, abnormal blood pressure patterns are highly prevalent in pediatric kidney transplant recipients. Our study demonstrated significantly higher prevalence of all ABP phenotypes under the 2022 guidelines compared to the 2014 guidelines. Furthermore, the significantly high prevalence of MH and isolated nocturnal hypertension, regardless of prior diagnosis, indicates that clinic blood pressures alone are insufficient to diagnose new-onset hypertension or to ensure treatment adequacy. The increased identification of ABPM abnormalities in those with elevated AASI using the 2022 guidelines underscores its role in identifying patients with increased cardiovascular risk. Based on our findings, we suggest that transplant centers consider incorporating 24-h ABPM into standard protocols to optimize the diagnosis and management of hypertension in the pediatric kidney transplant population.

## Supplementary Information

Below is the link to the electronic supplementary material.

Graphical abstract (PPTX 204 KB)

## Data Availability

Data is available from the authors upon request.
