# Supplementary material for: Impact of the 2022 ambulatory blood pressure monitoring guidelines on blood pressure phenotypes in pediatric kidney transplant recipients
Source: Pediatr Nephrol. 2026 Mar 10;41(8):2601–9. doi: 10.1007/s00467-026-07241-6 (PMC13337851; doi:10.1007/s00467-026-07241-6)
Supplement: Supplementary file 1 — Graphical abstract (PPTX 204 KB) [file 467_2026_7241_MOESM1_ESM.pptx]

## Slide 1
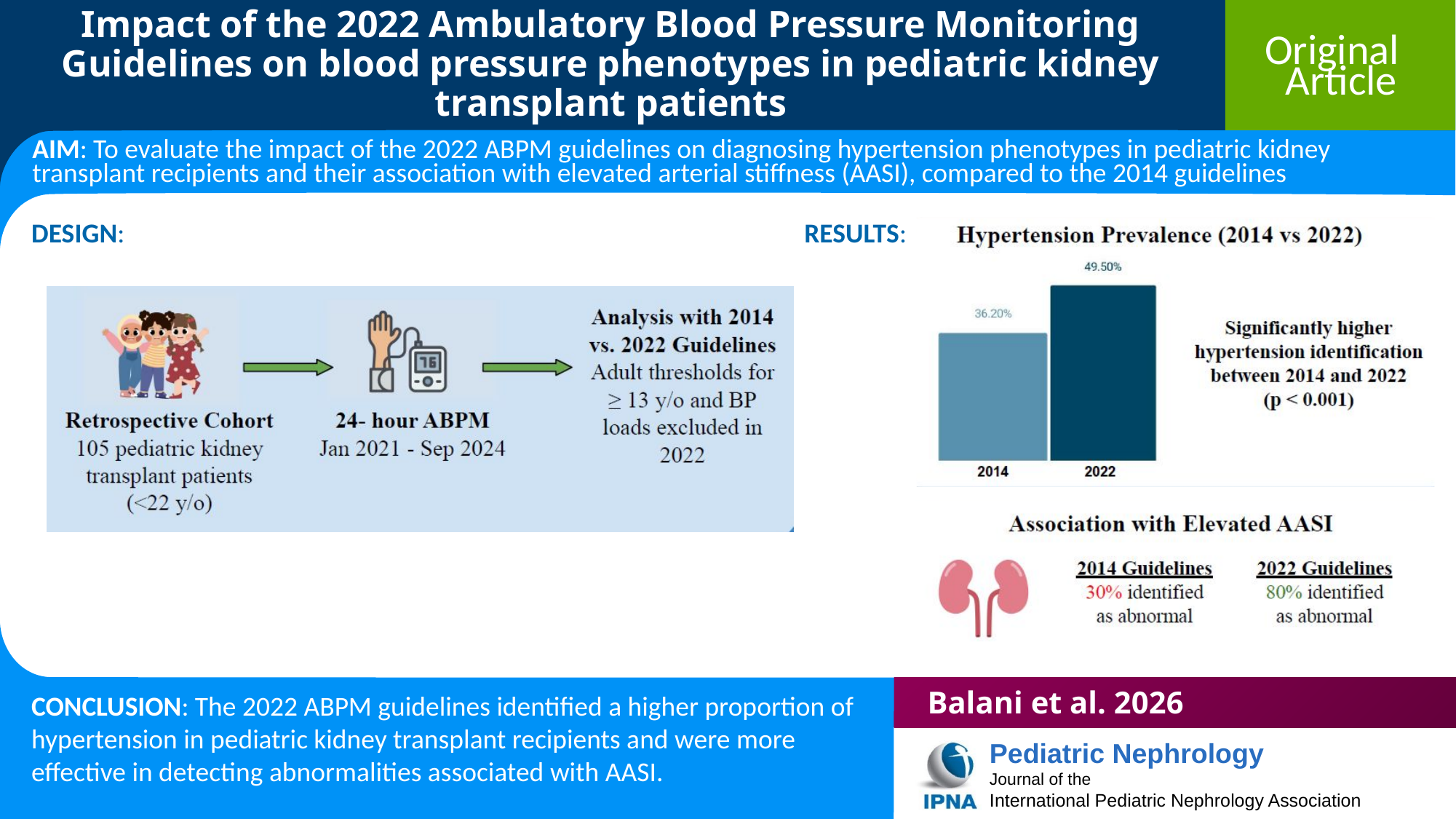

Impact of the 2022 Ambulatory Blood Pressure Monitoring Guidelines on blood pressure phenotypes in pediatric kidney transplant patients
AIM: To evaluate the impact of the 2022 ABPM guidelines on diagnosing hypertension phenotypes in pediatric kidney
transplant recipients and their association with elevated arterial stiffness (AASI), compared to the 2014 guidelines
DESIGN:
RESULTS:
Balani et al. 2026
CONCLUSION: The 2022 ABPM guidelines identified a higher proportion of hypertension in pediatric kidney transplant recipients and were more effective in detecting abnormalities associated with AASI.
